# Developing a Prognostic Information System for Personalized Care in Real Time

**DOI:** 10.5334/egems.266

**Published:** 2019-03-25

**Authors:** Tracy A. Lieu, Lisa J. Herrinton, Dimitri E. Buzkov, Liyan Liu, Deborah Lyons, Romain Neugebauer, Tami Needham, Daniel Ng, Stephanie Prausnitz, Kam Stewart, Stephen K. Van Den Eeden, David M. Baer

**Affiliations:** 1 The Permanente Medical Group, Kaiser Permanente Northern California, US; 2 Division of Research, Kaiser Permanente Northern California, US; 3 TPMG Technology Group, Kaiser Permanente Northern California, US; 4 Department of Oncology, Kaiser Permanente Oakland Medical Center, US

**Keywords:** Electronic health records, delivery of health care, organizational innovation, patient-centered care

## Abstract

**Context:**

Electronic medical records hold promise to transform clinical practice. However, technological and other barriers may preclude using them to guide care in real time. We used the Virtual Data Warehouse (VDW) to develop a tool that enables physicians to generate real-time, personalized prognostic information about survival after cancer.

**Case description:**

Patients with cancer often ask their oncologists, “Have you ever seen a patient like me?” To help oncologists answer this question, we developed a prototype Prognostic Information System (PRISM), a web-based tool that gathers data about the index patient from Kaiser Permanente’s clinical information systems, selects a historical cohort of similar patients, and displays the survival curve of the similar patients relative to key points in their treatment course.

**Findings and major themes:**

The prototype was developed by a multidisciplinary team with expertise in oncology, research, and technology. We have completed two rounds of user testing and refinement. Successful development rested on: (1) executive support and a clinical champion; (2) collaboration among experts from multiple disciplines; (3) starting with simple cases rather than ambitious ones; (4) extensive research experience with the Virtual Data Warehouse, related databases, and an existing query tool; and (5) following agile software development principles, especially iterative user testing.

**Conclusion:**

Clinical data stored in health care systems’ electronic medical records can be used to personalize clinical care in real time. Development of prognostic information systems can be accelerated by collaborations among researchers, technology specialists, and clinicians and by use of existing technology like the Virtual Data Warehouse.

## Context

Physicians and health systems are eager to reap the benefits of using the evidence that can be generated from electronic medical records (EMRs), yet are frustrated that more useful tools are not yet available [1]. More than 9 of 10 physician practices have EMRs, creating the potential to use data to inform clinical decisions and support learning health systems [2]. However, a gap remains between the promise of data science to transform health care and the limitations of implementing reliable user-tested tools in actual practice.

Oncologists are often asked by patients newly diagnosed with cancer, “Have you ever seen a patient like me?” This question may convey the patient’s need to be treated as an individual as well as concern about the oncologist’s experience with their particular cancer, especially in the current era of targeted therapy. In oncology and other specialties, physicians could benefit from a tool that rapidly identifies and analyzes a group of patients similar to the patient they are counseling. Providing data on the actual outcomes of similar patients would enable conversations that are more personalized and credible than those based solely on published literature [3]. The need for personalized information has led to the development of “patients like me” websites and tools that connect groups of patients based on key characteristics [4,5]. In parallel, physicians would value tools that can rapidly analyze and display outcomes in the course of their conversations with patients.

Attempts to create “patients like me” tools by organizations such as the MD Anderson Cancer Center and the American Society of Clinical Oncology have not yet succeeded despite large financial investments [6,7]. A major barrier has been the lack of comprehensive patient-level data over multi-year periods that cover the course of the patient’s disease trajectory and long-term outcomes including survival. A second major barrier is the technologic complexity of using electronic medical record data to create predictions rapidly enough to provide information to individual patients during clinical encounters. This case study describes how we developed a prototype that overcomes these barriers to provide physicians with real-time prognostic information tailored to individual patients for use during clinical encounters.

## Case Description

### Setting and Conception

This project was conducted by a multidisciplinary team within The Permanente Medical Group, which partners with Kaiser Foundation Health Plan to serve more than 4 million patients in Northern California. Our integrated health care system, Kaiser Permanente Northern California (KPNC), includes 21 medical centers and more than 100 oncologists. More than 17,000 patients in this population are newly diagnosed with cancer each year. Like all Kaiser Permanente regions, KPNC uses EPIC as the core of its EMR.

In 2015, a KPNC oncology leader (D.M.B.) requested that our organization’s Division of Research develop a tool to generate personalized information from our own health care databases for use in counseling cancer patients. In response, we created a prototype Prognostic Information System (PRISM) to enable physicians to generate personalized information for individual patients during real-time clinical encounters. PRISM generates personalized prognostic information by gathering key characteristics about the index patient, identifying a cohort of similar patients that are matched to the index patient on these characteristics, and displaying the survival curves of the similar patients.

### Data infrastructure

PRISM builds on many years of development and research use of the Virtual Data Warehouse (VDW), a data model developed by the Health Care Systems Research Network to facilitate public domain health and health services research [8]. The VDW was initiated during the 1990s with support from the Cancer Research Network and increasingly expanded and standardized over the years with support from many other federally-sponsored research networks. It provides an efficient, reliable, and convenient store of the data that are essential to our research activities. In KPNC, the VDW includes data on all diagnoses, treatments, and clinical outcomes for more than 9.6 million patients seen since 1996. Among the data elements relevant to oncology are cancer diagnoses, surgery, radiation, and intravenous and oral chemotherapy, and mortality data drawn from the KPNC Cancer Registry and multiple sources including pathology reports, diagnosis and treatment data, and multiple vital sources, including state death certificate data [8]. The VDW also incorporates a comorbidity score, the COPS2 score, We chose to use this comorbidity score because it better predicts outcomes than individual comorbidities and is simpler to display. It has been validated as an excellent predictor of mortality and has been updated every month for every KPNC member since 2010 [9].

**Figure 1:**
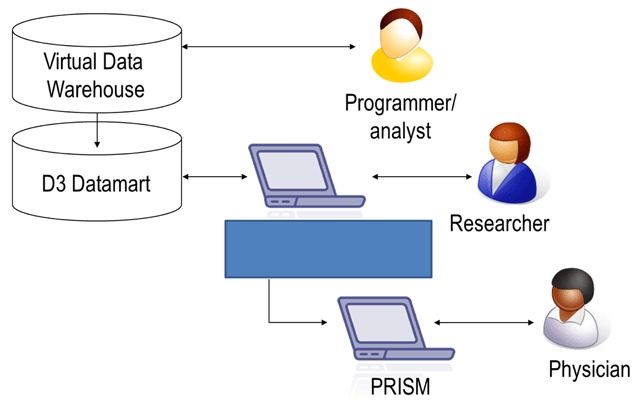
The Virtual Data Warehouse (VDW) of Kaiser Permanente Northern California, Division of Research Data on Demand (D3) Query Tool, and Prognostic Information System (PRISM).

The initial PRISM prototype relied on data architecture from the Division of Research Data-on-Demand (D3) tool (Figure 1), a web-based query tool implemented to increase the accessibility of data stored in the VDW [10,11]. The D3 tool was developed in 2013 by one of our computer scientists (D.E.B.). It enables authorized staff to access data and perform simple analyses through a graphical user interface, instead of writing complex scripting or programming code.

The D3 tool is a Java application with an HTML user interface supporting web browsers Internet Explorer and Chrome. The application and web services run on Apache Tomcat on a Windows VM server. The front-end application queries a back-end database via ANSI SQL. The database is a customized version of the VDW, transformed into an OLAP star schema in Teradata. Data are refreshed via monthly extract, transform, and load (ETL) running in a SAS container on a Sun Solaris server, pulling from various source data including the VDW, Epic Clarity, and other local data. The most current PRISM prototype is built in a .NET MVC framework, using Java to access the back-end database, which is an Oracle Exadata datamart composed of specific subsets of prospective and historical cancer patients.

### First Prototype: Diffuse B-Cell Lymphoma

We selected diffuse B-cell lymphoma for PRISM’s first prototype because its treatment is simple in involving only chemotherapy and not surgery or radiation. We devoted 12 months to creating a user interface and query structure for PRISM that focused on diffuse B-cell lymphoma but could be generalized to other cancer types. The team for the first prototype included researchers (T.A.L., L.J.H.), experts in data architecture and query programming (D.E.B., D.N.), a project manager (S.P.), and the oncology leader (D.M.B.).

The first PRISM prototype asked the user to specify the cancer type and stage, age range, sex, race/ethnicity, and COPS2 comorbidity score quartile. These variables were selected based on clinical experience. The user was then prompted to specify the treatments of interest. Using these inputs, PRISM selected similar patients from among all KPNC patients treated for diffuse large B-cell lymphoma. PRISM generated two screens with results: one with a survival curve for similar patients and one with a set of survival curves for all patients with diffuse B-cell lymphoma stratified by quartile of COPS2 comorbidity score.

We conducted user testing of this prototype with six oncologists. All testers felt that it was potentially useful, could envision using it to prepare for consultations with patients, and said it was worth developing further. The tests also identified issues and concerns. A key concern was that all six testers said they would like to use PRISM to choose treatments. Use of observational data to make treatment decisions is well documented to be fraught with complexity and potential bias due to confounding by indication and other problems [12,13]. For this reason, we did not design PRISM to help users select treatments or conduct ad-hoc comparative effectiveness analyses.

In addition, users asked that PRISM automatically select similar patients based on the known characteristics of an index patient, rather than asking the user to manually enter these specifications. One user expressed concern that if printed survival curves were handed to a patient, they might be compared with survival data available in the literature or offered by outside medical groups, leading to confusion or loss of trust. Survival following diffuse large B-cell lymphoma is relatively good, and users wanted the next prototype to address a cancer with worse survival, for which prognostic information would be more central to patient counseling.

### Second Prototype: Pancreatic Cancer

Based on the encouraging appraisal of the first prototype, we gained executive sponsorship and funding to develop a second. We chose pancreatic cancer because it has a low survival rate and the treatment has not changed dramatically in recent years We expanded the team to include an epidemiologist with expertise in pancreatic cancer (S.K.V.), as well as experts in technology (D.L., T.N., K.S., and others), a biostatistician (R.N.), and a data scientist (L.L.).

We developed the second prototype using lessons learned from the first. We aimed for the second prototype to be simple, comprehensible, valid, and rapid enough for an oncologist to use on a computer screen during a clinical interaction with a patient. We developed new approaches to (1) enabling PRISM to be used at different points in a patient’s clinical course, not only at diagnosis; (2) identifying variables for use in selecting similar patients; (3) developing the user interface for ease of use and comprehension; and (4) training users to not use PRISM to select treatments.

*Enabling use at different points in a patient’s clinical course*. The team’s oncologist asked for PRISM to predict survival at initial diagnosis and at other specific points during treatment. We diagrammed the possible treatment pathways for pancreatic cancer, including no treatment; surgery with or without adjuvant or neoadjuvant chemotherapy; radiation; and first-line, second-line, and third-line or higher-order palliative chemotherapy. We then designed PRISM to elicit input from the oncologist on which treatments the patient had completed, started, or planned. This input needed to be elicited from the oncologist because treatments that were planned would not yet be recorded in computerized data. We designed PRISM to select similar patients who had the same treatment history as the index patient, and to calculate survival from the start of that phase in treatment. Survival calculations are made at one of four time points: at diagnosis, at surgery, at the start of first-line palliative chemotherapy, or at the start of second-line palliative chemotherapy. For example, given an index patient who has had surgery and is now planning first-line palliative chemotherapy, PRISM selects similar patients who have had surgery and who had first-line palliative chemotherapy, and calculates survival from the start of that chemotherapy.

*Selecting similar-patient groups*. To characterize similar patient groups, we first identified 4,431 patients with pancreatic cancer diagnosed within KPNC between 2005 and 2014. We classified these into six tumor type subgroups, which had sample sizes ranging from 3,499 (for ductal adenocarcinoma) to 5 (for acinar cell carcinoma). We further divided these subgroups by cancer stage. For each of the possible treatment pathways, for tumor type/stage subgroups with 100 patients or more, we used proportional hazards regression modeling to identify variables to be used to personalize prognosis. Constructing these models enabled us to identify predictors associated with survival for patients who had different treatment pathways, so that PRISM could use these variables to select the best similar-patient group and provide more personalized prognostic information than would be available from published literature. We tested predictors that had been identified in previous studies of pancreatic cancer [14,15,16], conducted iterative modeling to select variables that were independently and significantly associated with survival, and constructed one model for each possible treatment pathway (e.g., no treatment; surgery followed by no treatment; surgery followed by first-line palliative chemotherapy; first-line palliative chemotherapy alone; second-line or higher palliative chemotherapy, etc.). We also constructed models for patients who had not yet decided on specific treatment plans; these models included all patients, including those with and without the treatment. The variables used as predictors in the final models included age, sex, comorbidity score, weight loss of 5 percent or more the past 12 months, and laboratory tests (aspartate aminotransferase, CA 19–9, and percentage lymphocytes); not every predictor was used in every model.

It is important to note that PRISM uses deterministic business rules to select similar patients from the KPNC population, then creates a survival curve with 95 percent confidence limits for the similar patients. It does not calculate the survival curves by running a parametric statistical model. The primary purpose of our proportional hazards modeling was to select predictors to be used to personalize prognosis.

*Developing the user interface*. The TPMG Technology Group team developed a professional-grade user interface that a physician could show to a patient during a clinic visit. The user interface is designed to increase patients’ understanding of their survival outcomes and their confidence in decisions about their care. PRISM is designed to offer physician-users simplicity of use as well as the ability to tailor inputs individually and view results in ways that inform and assist conversations with patients.

PRISM asks the oncologist to input the patient’s medical record number, then auto-populates the patient’s age, sex, COPS2 comorbidity score, weight change in the past 90 days, and recent laboratory values. It then prompts the user to input tumor type, stage at diagnosis, treatments completed or in progress, and treatments planned.

After the index patient is specified, PRISM selects similar patients and displays the ranges of their variables. For example, for a 60-year-old index patient, we selected a default age range for similar patients of 55–65 years based on our descriptive analyses and proportional hazards models. The types of data gathered and processed by PRISM come from the VDW and other sources (Table 1). Some data sources are refreshed daily while others are refreshed less frequently.

**Table 1:** Types of Data Processed by the Prognostic Information System (PRISM) Prototype for Pancreatic Cancer, with the Requirements for Timeliness of Capture.

Data type/variable	Data source for index patient	Data source for similar patients	Lag time	Comments

**Variables autopopulated by PRISM**				

Age, sex	Clarity	VDW	For Clarity, one day For VDW, one month	
COPS2 comorbidity score	VDW*	VDW*	One month	
Weight change in last 90 days Laboratory results	Clarity	VDW	For Clarity, one day For VDW, one month	User can enter today’s weight or laboratory results, if available
Year of pancreatic cancer diagnosis	VDW	KPNC Cancer Registry and VDW	For VDW, one day For KPNC Cancer Registry, six months (with monthly updates)	User can enter the diagnosis year for patients who do not have a diagnosis before their first visit
**Variables entered by the oncologist**				

Tumor type	Pathology	KPNC Cancer Registry	For KPNC Cancer Registry, six months (with monthly updates)	Could be derived from pathology reports via natural language processing but difficult to do this in real time
Stage at diagnosis	Oncologist	KPNC Cancer Registry	NA	Complex variable; may eventually become available more routinely via a structured staging tool
Treatments completed, in progress, and planned	Oncologist and patient	VDW	For VDW, one month	
**Outcome variables**				

Death	Not applicable	VDW	Three months (with quarterly updates)	Sources of mortality data are KPNC clinical and administrative data, Social Security Death Master files, California State death certificate data

VDW: Virtual Data Warehouse. KPNC: Kaiser Permanente Northern California. NA: Not applicable. * Generated by another KPNC department in the Integrated Data Repository and shared with the VDW.

A special feature of PRISM is that it allows the user to modify the size of the similar-patients cohort by editing the ranges of selected matching variables (e.g., the age range). It also displays the sample size of the similar-patient group. Thus, PRISM enables the physician-user to trade off statistical precision (which is obtained by using a larger cohort) with personalization (which is achieved by using a more precise definition of similarity between patients).

**Figure 2:**
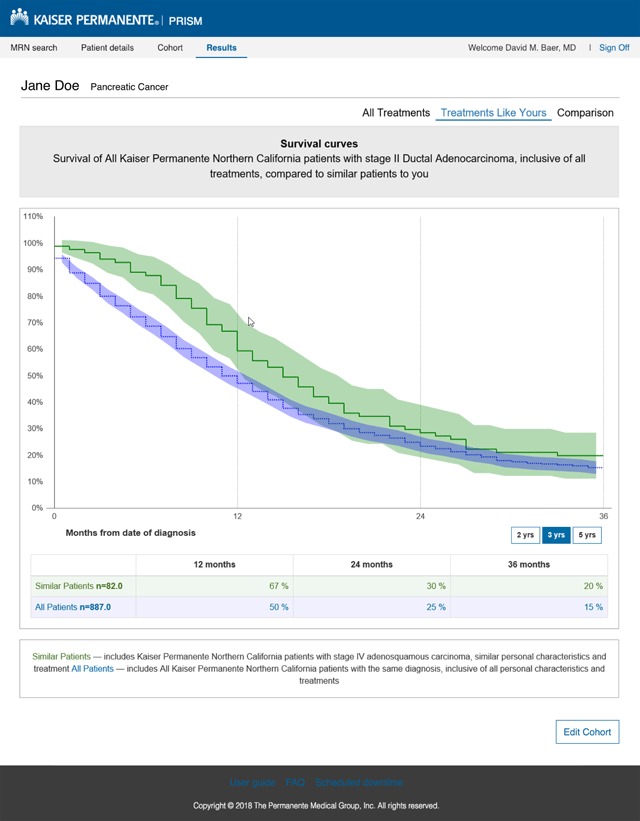
Results Screen Presented by the Prognostic Information System (PRISM).

The results screen displays a survival curve and 95 percent confidence interval for the similar-patient group that can be easily interpreted by the doctor and shared with the patient (Figure 2). PRISM also generates a screen that compares the survival curve of similar patients with that of all patients with the same tumor type and stage. This is to help the oncologist explain why the patient’s prognosis might be different from published estimates for the general population, for example on the American Cancer Society website [17]. PRISM also generates screens with survival curves stratified by age and COPS2 comorbidity score.

*Training users to not use PRISM to select treatments*. Because PRISM was not designed to evaluate counterfactual survival curves under various therapy decisions, attempts to use it for ad-hoc comparative effectiveness analyses are prone to confounding by indication and are at high risk of yielding erroneous causal inferences. Thus, we designed a special module to train users to not use PRISM to conduct ad-hoc comparative effectiveness analyses to guide treatment selection.

The training module uses the example of autologous bone-marrow transplantation of breast cancer [18]. We selected this example because it received substantial media attention and is familiar to most oncologists. In the 1980s, researchers used the results of observational studies to claim that autologous bone-marrow transplantation increased survival for breast cancer patients [19,20]. Based on these studies and the associated media attention, autologous bone-marrow transplantation became the standard of care. It was not until the early 2000s that randomized trials clearly demonstrated that such transplantation was ineffective and harmful [21,22]. The earlier findings that transplantation was associated with benefit stemmed from healthier patients with more comprehensive staging and less extensive disease having been selected to receive transplantation. Our training module, placed within the user introduction materials, is designed to show that using PRISM to make treatment decisions could lead to the same types of errors made by the proponents of autologous bone-marrow transplantation for breast cancer.

*User testing of the pancreatic cancer prototype*. We have recently completed user testing of the second prototype’s user interface with five oncologists, two of whom were testers of the first prototype. All users could imagine using PRISM in clinical practice and were generally supportive of continued development. However, all identified specific problems with tool’s design. Their overall reactions ranged from enthusiastic to mildly skeptical. Users suggested major areas for improvement, noting that they: (1) felt burdened when they had to input data they thought could be auto-populated, such as laboratory values; (2) became confused when they were presented with the COPS2 comorbidity score and the percent weight change within the last 90 days without clear documentation of how these data had been derived; (3) found the survival curves stratified by comorbidity and age less useful than those that showed similar patients and all patients with the same tumor type and stage; and (4) wanted clarification of the specific factors that made their patient’s predicted survival different from other patients with the same tumor type and stage.

Our next steps are to refine PRISM’s user interface, algorithms, and back-end architecture, connect the user interface and back ends, and conduct at least one more round of user testing. Technical requirements include (1) creating a translation layer that enables the user interface to interact with the existing back-end VDW query tool data architecture; (2) adding secure sign-on and addressing other risks; (3) identifying a site to host the user introduction and user guide; and (4) creating a place to store data input by the user.

Validation of PRISM will use hypothetical cases to test a priori hypotheses about how survival curves should behave. In addition, we will compare the results of PRISM with the results of statistical analyses performed by the research team. We also will need to make decisions about how to handle situations in which the numbers of patients in a given subgroup become small. Once we are confident that PRISM is stable, valid, and user-friendly, we plan to introduce it to selected clinical leaders and early adopters for evaluation it in clinical practice.

## Findings and Major Themes

Successful development of the PRISM prototype has addressed seven dimensions described in a strategic innovation framework, including strategic alignment, insight about emerging trends and customer (physicians’ and patients’) needs, and leveraging and extending existing assets (Table 2) [23]. Specifically, this project’s progress has relied on (1) executive support and a clinical champion; (2) collaboration among experts from multiple disciplines; (3) starting with simple cases rather than ambitious ones; (4) extensive research experience with the Virtual Data Warehouse, related databases, and an existing query tool; and (5) following agile software development principles, especially iterative user testing.

**Table 2:** Dimensions of Strategic Innovation [23] Applied in Development of the Prognostic Information System (PRISM).

Dimension of Strategic Innovation		How Applied in PRISM Development

1.	Managed innovation (combining traditional and non-traditional approaches)	The process combines traditional statistical and research methods with modern technology development processes.
2.	Strategic alignment (building support)	The development work is being supported by multiple stakeholders including executives and teams from multiple disciplines.
3.	Industry foresight (understanding emerging trends)	The project is aligned with emerging trends in health care, in which data science and informatics are being used to personalize care for individual patients.
4.	Consumer/customer insight (understanding articulated and unarticulated needs)	PRISM is designed to meet the needs described by the oncologist leader of the team, as well as the oncologist users. It also addresses the objectives of executive sponsors to fully use our data to optimize patient care.
5.	Core technologies and competencies (leveraging and extending existing assets)	PRISM leverages our skills in research, technology, and clinical care, and builds on areas of synergy among these disciplines.
6.	Organizational readiness (ability to take action)	The oncologists who are prospective users of PRISM express readiness to use it; there is cultural, process, and structural readiness to test and implement PRISM.
7.	Disciplined implementation (managing the path from inspiration to implementation)	Each step in PRISM’s development has been managed to achieve a goal feasible within 6 to 12 months, with iterative adjustments as the team has learned about the needs that must be addressed to achieve implementation.

Executive support has been a key element in the development of PRISM because the substantial effort required from the research and technology teams could not have been invested without sponsorship and encouragement from these high-level leaders. The clinical champion has been essential for articulating the vision and guiding the team through key decisions to ensure PRISM’s fit into real-life workflows. He also recruited other oncologists to test PRISM, and he cultivated interest in PRISM among these colleagues.

The PRISM team brings together experts from multiple disciplines, including oncology, computer software development, user interface design, epidemiological methods, data science, and biostatistics. Bringing these experts into face-to-face meetings shortened the time needed for development and enabled the team to troubleshoot complex issues. By working with the oncologist as the product owner, the multidisciplinary team has assured that PRISM addresses the needs of end-users as best possible within existing data structures.

The relatively simple use cases used for the first and second PRISM prototypes enabled the project team to learn in a focused fashion as we created a common understanding and common set of approaches. Use of simple cases also enabled more rapid development of prototypes and simplified user testing.

Our team’s extensive experience with the VDW and other research databases has been another success factor. The VDW makes it possible to run analytic queries in seconds. The fact that our oncologists create the data in the EMR, which subsequently populate the VDW and PRISM, gives them an inherent understanding of the information that PRISM produces. Experience building the D3 query tool taught the team key skills for assembling PRISM’s back-end architecture.

Our team has followed agile software development principles (Table 3), which emphasize close collaboration among end-users, developers, and researchers to assure the design satisfies the end-user; frequent demonstrations of PRISM and iterative development to enable the end-user to revise business requirements; face-to-face meetings; and consideration for changing the schedule as needs evolve [24,25]. For example, our initial plan was crafted by researchers who planned a 24-month timeline with 9 months of development and 15 months of research. Now that the research and technology teams have worked together, we realize that the project needs 15 months for development. We have reiterated and clarified that the project’s focus is to produce a usable prototype and to produce knowledge that generalizes to the next phase of this tool.

**Table 3:** Agile Software Development Concepts [24] Applied in Development of the Prognostic Information System (PRISM)*.

Agile Software Concept		How Applied in PRISM Development

1.	Iterative and incremental approaches	Work is broken into small increments that help limit the initial planning and design. Several iterations of the user interface have been tested.
2.	Efficient and face-to-face communication	The multidisciplinary team holds face-to-face meetings as needed to address the evolving requirements of the project. The oncologist and lead researcher serve as the product owners and are available whenever the developers have questions.
3.	Adaptive approaches with short feedback loops	The project plan changes each month to incorporate the activities needed to develop PRISM, given the currently available data sources and technological environment. The research component of the project has been reduced to make time for additional iterations of PRISM’s development.
4.	Quality focus	Quality assurance will be conducted through validation testing before PRISM is released for oncologists to use with patients. Safety and security needs are also being addressed through user training and software configuration.
5.	Self-organizing team of motivated individuals	The project is organized by a multidisciplinary team of highly experienced individuals. The team has adjusted its approaches and the project plan at regular intervals to enhance the effectiveness of collaborative interactions and time invested.

Important questions remain about how this tool will be used in actual patient encounters. PRISM is one of many ongoing efforts to create tools for prognosis or prediction. Adoption of such tools in actual practice has remained limited, in part because they are sometimes developed without a clear picture of how they will influence real-life decision processes [26]. Experience in our setting suggests that prediction tools may succeed best when their development is initiated and promoted by clinicians [27,28]. Although the oncologists who have tested PRISM have described how it could be useful in conversations with patients, empirical evaluation in clinician-patient interactions is needed to elucidate its actual benefits and limitations. User acceptability testing with patients will be an important next step. Such testing could compare alternative methods of offering the prognostic information, for example via one-year survival estimates versus survival curves.

The methods used to create this tool are generalizable to other health care systems, especially those with VDWs. All components of the D3 tool and PRISM prototypes use widely available, industry standard application languages, operating environments, and analytical/query methods. Most of the data elements required exist in the VDW, which has files for tumor characteristics and chemotherapy, but specific information such as tumor type may need to be drawn from a cancer tumor registry.

The cost of PRISM’s development to date is estimated at several hundred thousand dollars, almost exclusively for personnel effort. This is far less than the tens of millions of dollars reported for other oncology decision support systems in development [6]. However, PRISM is a simpler tool than others because it does not attempt to recommend a best treatment, but instead shows prognostic information for a specified course of treatment. In addition, the foundation for PRISM in our setting had already been built via our existing VDW data infrastructure and the D3 query tool.

## Conclusion

Web-based tools can enable physicians to use EMR data to generate personalized prognostic information for clinical care in real time. The process of developing such tools benefits from strategic innovation and agile software development methods. Progress can be accelerated by collaborations among researchers, technology specialists, and clinicians, and structures like the Virtual Data Warehouse.
